# Orf Nodule with Erythema Multiforme during a Monkeypox Outbreak, France, 2022

**DOI:** 10.3201/eid2904.230058

**Published:** 2023-04

**Authors:** Charlotte Cavalieri, Anne-Sophie Dupond, Audrey Ferrier-Rembert, Olivier Ferraris, Timothée Klopfenstein, Souheil Zayet

**Affiliations:** Nord Franche-Comté Hospital, Trévenans, France (C. Cavalieri, A.-S. Dupond, T. Klopfenstein, S. Zayet);; Institut de Recherche Biomédicale des Armées, Brétigny-sur-Orge, France (A. Ferrier-Rembert, O. Ferraris)

**Keywords:** monkeypox, orf nodule, erythema multiforme, parapoxvirus, viruses, PCR, France

## Abstract

A 26-year-old patient in France who worked as a butcher sought care initially for erythema multiforme. Clinical examination revealed a nodule with a crusty center, which upon investigation appeared to be an orf nodule. Diagnosis was confirmed by PCR. The patient was not isolated and had a favorable outcome after basic wound care.

Orf nodule is a rare viral zoonosis attributable to an infection caused by a parapoxvirus (*1*). It is transmitted to humans by contact with sheep or goats that are affected by contagious ecthyma. The term orf is used to designate contagious ovine pustular dermatitis. In infected sheep and goats, infection most often results in perioral and perinasal ulcerations but occasionally also in a generalized pustular rash.

The most at risk for exposure are persons working in the meat sector, such as farmers or butchers (*1*). Farmers and those who maintain animal herds are often familiar with the condition and do not seek medical attention. Clinical visits for orf may therefore be more common among nonfarmers. Incidence often peaks at the time of religious festivals, when sheep are traditionally sacrificed, and the incubation time is approximately 1 week (*2*). The diagnosis is basically clinical and can be confirmed with PCR (*3*). Orf nodules may resemble mpox lesions, but unlike mpox, orf is not transmitted from human to human. Routine precautions in clinical settings are sufficient, and patients are not recommended to isolate.

In August 2022, a 26-year-old man with no notable medical history visited an emergency department for disseminated skin lesions predominantly in acral areas. The patient lives with his wife and children in Franche-Comté, France, and works as a butcher. He denied extraconjugal sex, including sex with men, and using illicit drugs, and he had not traveled recently. Neither fever nor contagion was reported. His attending physician prescribed local antibiotics (fucidin acid) and oral antibiotic drugs (amoxicillin/clavulanate), with no effect.

Clinical examination revealed symmetric maculopapular lesions predominantly on the palms and foot, with purplish center and pinkish halo (target shaped lesions), typical of erythema multiforme (Figure, panel A, B). Results of respiratory and neurologic examinations were unremarkable. A nodule with a necrotic pustule center was surrounded by a grayish-white edematous crown on the left index finger, suggesting a lesion of orf nodule more than a monkeypox infection (Figure, panel C). This lesion appeared 72 hours before the disseminated cocoon lesions, according to the patient.

**Figure:**
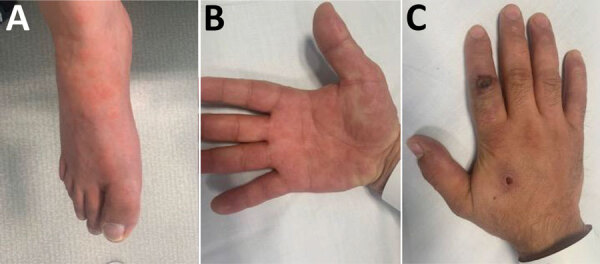
Orf virus infection in a 26-year-old man after contact with slaughtered sheep and goats, France, 2022. A, B) Target lesion characteristic of erythema multiforme predominant on the foot (A) and hand (B). C) Nodule caused with a necrotic pustule center surrounded by a grayish-white edematous crown on the left index finger. Lesion was swabbed and tested by using a parapoxvirus PCR test.

Laboratory findings showed white cell count of 9.2 G/L (reference range 4–10 G/L) but lymphopenia of 880/mm^3^ (reference range 1,500–4,000/mm^3^). C-reactive protein was moderately high at 10 mg/L (reference range <5 mg/L); liver function was normal. Results of PCR for herpes simplex viruses 1 and 2 on skin biopsy were negative, as were serologic tests for *Mycoplasma pneumoniae*, HIV, and hepatitis B and C viruses. Because of the ongoing mpox outbreak, PCR for monkeypox virus was, performed after simple swabbing on the pustule, but results were negative.

Parapoxvirus PCR was performed by swab of nodule (on the left index finger) (Figure, panel C). The sample was sent to the National Reference Center for Orthopoxvirus Expert Laboratory (*3*). The laboratory used 2 real-time PCRs to confirm the diagnosis of orf nodule. The first assay detected parapoxvirus on the basis of the B2L and J6R genes; the second assay detected orf virus on the basis of the V22R and J6R genes. The patient was discharged with basic wound care, discontinuation of antibiotics, and a follow-up appointment 1 week later. At follow-up, erythema multiforme had disappeared and the nodule clinical regressed.

In typical forms, orf nodule is a skin lesion, unique to the area of inoculation, in particular the right fingers and forearm. A macular lesion appears and rapidly becomes papulovesicular, then nodular, surrounded by an inflammatory halo. Other forms include botriomycoid, angiomatous, or keratoacanthoma-like. The lesion generally heals without complication with antiseptic treatment in 4–6 weeks but in rare cases, erythema multiforme develops (*2*,*4*,*5*).

How orf virus induces erythema multiforme is not clearly understood. Other viral infections like herpes simplex viruses can also trigger hypersensitivity reaction because of release of T cells triggered by ether viral mimicry of host proteins or release of viral proteins from cells containing viral DNA fragments (*1*).

In this case, the patient experienced inaugural erythema multiforme and secondarily a suspected lesion of orf nodule in a period when monkeypox virus infection was endemic (i.e., >4,000 orthopoxvirus infections have been reported in France since May 2022) (*6*). Lesions of monkeypox and orf can be similar, but the manifestations are sufficiently distinctive (*7*,*8*). In this case, orf virus infection was suspected because of the patient’s occupational exposure and clinical compatible skin lesions (e.g., single pustular lesion and erythema multiforme aspect on the rest of the body and the absence of systemic symptoms) (*9*); infection was diagnosed with positive parapoxvirus PCR test (*3*). However, an unusual recent case in Portugal involved monkeypox infection after a needle stick injury (*10*). The patient had a solitary pustular lesion of the finger, similar to our patient, but that lesion was painful, and the clinical picture was completed with the appearance of diffuse vesicles and systemic symptoms.

This case highlights the importance of collecting a careful history at the time of patient care, including collection of exposures to possible zoonoses. Those measures are warranted to avoid unnecessary isolation and treatment and to enable appropriate infection control measures.
